# *N*,*N*-Diethyl­anilinium 5-(2,4-dinitro­phen­yl)-2,6-dioxo-1,2,3,6-tetra­hydro­pyrimidin-4-olate

**DOI:** 10.1107/S160053681204874X

**Published:** 2012-12-05

**Authors:** Doraisamyraja Kalaivani, Govindan Mangaiyarkarasi

**Affiliations:** a PG and Research Department of Chemistry, Seethalakshmi Ramaswami College, Tiruchirappalli 620 002, Tamil Nadu, India

## Abstract

The asymmetric unit of the title mol­ecular salt, C_10_H_16_N^+^·C_10_H_5_N_4_O_7_^−^ (trivial name: *N*,*N*-diethyl­anilinium 2,4-dinitro­phenyl­barbiturate), comprises two anion–cation units. In the anions, the dinitro­phenyl ring and the mean plane of the barbiturate ring [planar to within 0.011 (2) and 0.023 (2) Å in the two anions] are inclined to one another by 41.47 (9) and 45.12 (9)°. In the crystal, the anions are linked *via* strong N—H⋯O hydrogen bonds, forming chains propagating along [10-1]. Within the chains, adjacent inversion-related anionic barbiturate entities are joined through *R*_2_^2^(8) ring motifs. The cations are linked to the chains *via* N—H⋯O hydrogen bonds. The chains are linked *via* a number of C—H⋯O inter­actions, forming a three-dimensional structure.

## Related literature

For the crystal structures of related barbiturates, see: Kalaivani & Malarvizhi (2009 ▶); Buvaneswari & Kalaivani (2011*a* ▶,*b* ▶); Kalaivani *et al.* (2012 ▶); Babykala & Kalaivani (2012 ▶). For the biological activity of barbiturates, see: Hueso *et al.* (2003 ▶); Kalaivani *et al.* (2008 ▶); Tripathi (2009 ▶); Kalaivani & Buvaneswari (2010 ▶).
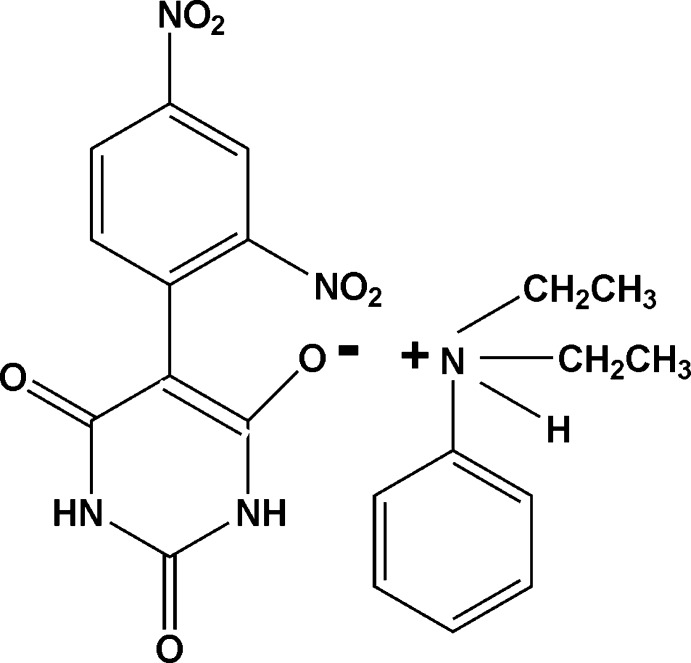


## Experimental

### 

#### Crystal data

C_10_H_16_N^+^·C_10_H_5_N_4_O_7_^−^
*M* *_r_* = 443.42
Triclinic, 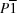

*a* = 8.7260 (2) Å
*b* = 14.2930 (3) Å
*c* = 18.1080 (5) Å
α = 106.712 (1)°
β = 96.490 (1)°
γ = 97.667 (1)°
*V* = 2116.27 (9) Å^3^
*Z* = 4
Mo *K*α radiation
μ = 0.11 mm^−1^
*T* = 293 K
0.30 × 0.30 × 0.25 mm

#### Data collection

Bruker Kappa APEXII CCD diffractometer
Absorption correction: multi-scan (*SADABS*; Bruker, 2004 ▶) *T*_min_ = 0.944, *T*_max_ = 0.996
36083 measured reflections
7482 independent reflections
5563 reflections with *I* > 2σ(*I*)
*R*_int_ = 0.029

#### Refinement

*R*[*F* ^2^ > 2σ(*F* ^2^)] = 0.042
*wR*(*F* ^2^) = 0.122
*S* = 1.02
7482 reflections
601 parameters
6 restraints
H atoms treated by a mixture of independent and constrained refinement
Δρ_max_ = 0.39 e Å^−3^
Δρ_min_ = −0.21 e Å^−3^

### 

Data collection: *APEX2* (Bruker, 2004 ▶); cell refinement: *APEX2* and *SAINT* (Bruker, 2004 ▶); data reduction: *SAINT* and *XPREP* (Bruker, 2004 ▶); program(s) used to solve structure: *SIR92* (Altomare *et al.*, 1993 ▶); program(s) used to refine structure: *SHELXL97* (Sheldrick, 2008 ▶); molecular graphics: *ORTEP-3* (Farrugia, 2012 ▶) and *Mercury* (Macrae *et al.*, 2008 ▶); software used to prepare material for publication: *SHELXL97*.

## Supplementary Material

Crystal structure: contains datablock(s) global, I. DOI: 10.1107/S160053681204874X/su2535sup1.cif

Structure factors: contains datablock(s) I. DOI: 10.1107/S160053681204874X/su2535Isup2.hkl

Supplementary material file. DOI: 10.1107/S160053681204874X/su2535Isup3.cml

Additional supplementary materials:  crystallographic information; 3D view; checkCIF report

## Figures and Tables

**Table 1:** Hydrogen-bond geometry (Å, °)

*D*—H⋯*A*	*D*—H	H⋯*A*	*D*⋯*A*	*D*—H⋯*A*
N3—H3*A*⋯O7^i^	0.89 (2)	2.00 (2)	2.878 (2)	172 (2)
N4—H4*A*⋯O14^ii^	0.87 (2)	1.93 (2)	2.802 (2)	172 (2)
N7—H7*A*⋯O13^iii^	0.88 (2)	2.06 (2)	2.931 (2)	175 (2)
N8—H8*A*⋯O6^iv^	0.89 (2)	1.98 (2)	2.852 (2)	164 (2)
N9—H9*A*⋯O12	0.90 (2)	1.83 (2)	2.726 (2)	176 (1)
N10—H10*A*⋯O5^v^	0.92 (2)	1.69 (2)	2.598 (3)	166 (2)
C12—H12⋯O4^vi^	0.93	2.52	3.451 (3)	174
C26—H26⋯O12	0.93	2.59	3.272 (3)	131
C26—H26⋯O13^iii^	0.93	2.56	3.281 (3)	135
C29—H29*B*⋯O11	0.97	2.57	3.215 (3)	124
C38—H38*A*⋯O7^i^	0.96	2.52	3.484 (3)	177

Symmetry codes: (i) 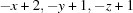; (ii) 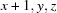; (iii) 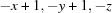; (iv) 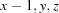; (v) 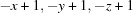; (vi) 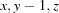.

**Fig. 1.**
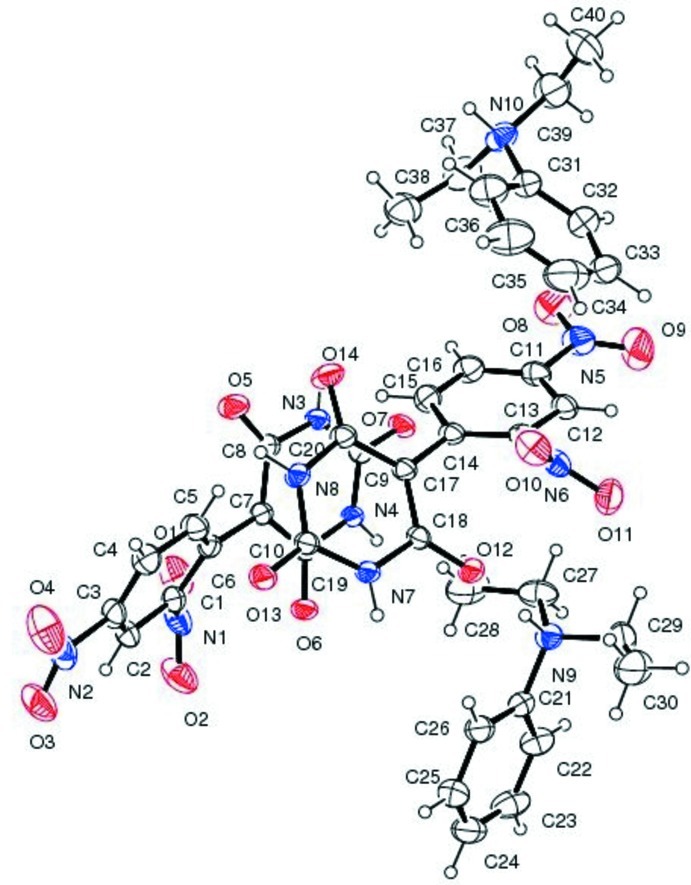
A view of the molecular structure of the title compound, with the numbering scheme. Displacement ellipsoids are drawn at the 30% probability.

**Fig. 2.**
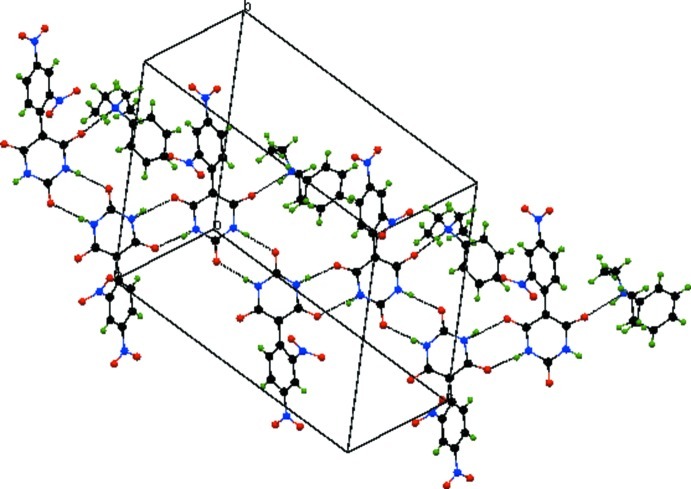
A partial view of the crystal packing of the title compound with the N-H···O hydrogen bonds shown as dashed lines (see Table 1 for details).

## supplementary crystallographic information

### Comment

The methylene group of barbituric acid [a pyrimidine derivative] is flanked on both sides by the electron-withdrawing carbonyl groups which makes the hydrogen atoms highly acidic. These acidic H atoms have been targeted by our group in the preparation of a number of extraordinarily stable barbiturates (Kalaivani & Malarvizhi, 2009; Buvaneswari & Kalaivani, 2011*a*; Kalaivani *et al.*, 2012; Babykala & Kalaivani, 2012). We have reported on the crystal structure of a barbiturate related to the title molecular salt but derived from 1-chloro-2,4,6-trinitrobenzene (TNCB) and barbituric acid in the presence of *N*,*N*-diethylaniline (I) (Buvaneswari & Kalaivani, 2011*b*). Herein we report on the crystal structure of the new title molecular salt obtained from 1-chloro-2,4-dinitrobenzene (DNCB) and barbituric acid in the presence of N,*N*-diethylaniline, (II).

Unlike the asymmetric unit of the related reported barbiturate (I), which comprises of only one anion and cation moieties, the asymmetric unit of the barbiturate of the title compound (II) is composed of two cations and two anions (Fig. 1). Contrary to the barbiturate of TNCB (I), which crystallized in the monoclinic space group P2_1_/c, the title compound (II) crystallized in the triclinic space group P1.

In the crystal of (II), the anions are linked via N—H···O hydrogen bonds (Table 1 and Fig. 2), forming chains along direction [1 0 -1]. This linkage and the *R*_2_^2^(8) ring motifs formed between inversion-related barbiturate residues contributes considerably to the extraordinary stability of the title molecular salt. The cations are linked to the chains via N-H···O hydrogen bonds (Table 1 and Fig. 2). There are C-H···O interactions present (Table 1) but no π-π stacking interactions between the *N*,*N*-diethylaniline and 2,4-dinitrophenyl ring moieties.

As barbiturates are employed in the treatment of neurological disorders (Hueso *et al.*, 2003; Kalaivani *et al.*, 2008; Tripathi, 2009; Kalaivani & Buvaneswari, 2010), the non-bonding interactions of the present investigation may help to understand the mechanistic aspects of the physiological action of barbiturates.

### Experimental

Analytical grade 1-chloro-2,4-dinitrobenzene (2.02 g, 0.01 mol) was dissolved in 20 ml of absolute alcohol. Barbituric acid (1.28 g, 0.01 mol) was also dissolved in 30 ml of absolute alcohol separately. These two solutions were then mixed well. To this mixture, ca. 4 ml of *N*,*N*-diethylaniine (0.03 mol) was added and shaken well for 5–6 hrs. The slightly turbid solution obtained was filtered and kept as such at room temperature. After a period of four weeks, dark shiny maroon red coloured crystals of the title salt crystallized out from this solution. The crystals were filtered and washed well with 30 ml of dry ether. The crystals were then powdered and washed with 5 ml of absolute alcohol to remove the unreacted reactants and finally with 25 ml of dry ether. The pure powder was then recrystallized from hot ethanol (M.p: 481 K; yield: 80%). Good quality single crystals, suitable for X-ray diffraction studies, were obtained by slow evaporation of a solution in ethanol at room temperature. The crystals obtained were non-hygroscopic and extraordinarily stable at room temperature.

### Refinement

The N-bound H atoms were located in a difference electron density map and refind with a N-H distance restraint of 0.90 (2) Å. The C-bound hydrogen atoms were placed in calculated positions and refined as riding atoms: C—H = 0.93, 0.97 and 0.96 Å for CH, CH_2_ and CH_3_ H atoms, respectively, with *U*_iso_(H) = k × *U*_eq_(C), where k = 1.5 for methyl H atoms and = 1.2 for other H atoms.

### Crystal data

**Table:**

C_10_H_16_N^+^·C_10_H_5_N_4_O_7_^−^	*Z* = 4
*M**_r_* = 443.42	*F*(000) = 928
Triclinic, *P*1	*D*_x_ = 1.392 Mg m^−^^3^
Hall symbol: -P 1	Mo *K*α radiation, λ = 0.71073 Å
*a* = 8.7260 (2) Å	Cell parameters from 5648 reflections
*b* = 14.2930 (3) Å	θ = 2.4–24.5°
*c* = 18.1080 (5) Å	µ = 0.11 mm^−^^1^
α = 106.712 (1)°	*T* = 293 K
β = 96.490 (1)°	Block, red
γ = 97.667 (1)°	0.30 × 0.30 × 0.25 mm
*V* = 2116.27 (9) Å^3^	

### Data collection

**Table:**

Bruker Kappa APEXII CCD diffractometer	7482 independent reflections
Radiation source: fine-focus sealed tube	5563 reflections with *I* > 2σ(*I*)
Graphite monochromator	*R*_int_ = 0.029
ω and φ scan	θ_max_ = 25.1°, θ_min_ = 1.6°
Absorption correction: multi-scan (*SADABS*; Bruker, 2004)	*h* = −10→10
*T*_min_ = 0.944, *T*_max_ = 0.996	*k* = −17→16
36083 measured reflections	*l* = −20→21

### Refinement

**Table:**

Refinement on *F*^2^	Primary atom site location: structure-invariant direct methods
Least-squares matrix: full	Secondary atom site location: difference Fourier map
*R*[*F*^2^ > 2σ(*F*^2^)] = 0.042	Hydrogen site location: inferred from neighbouring sites
*wR*(*F*^2^) = 0.122	H atoms treated by a mixture of independent and constrained refinement
*S* = 1.02	*w* = 1/[σ^2^(*F*_o_^2^) + (0.0554*P*)^2^ + 0.687*P*] where *P* = (*F*_o_^2^ + 2*F*_c_^2^)/3
7482 reflections	(Δ/σ)_max_ < 0.001
601 parameters	Δρ_max_ = 0.39 e Å^−^^3^
6 restraints	Δρ_min_ = −0.21 e Å^−^^3^

### Special details

**Table:**

Geometry. Bond distances, angles etc. have been calculated using the rounded fractional coordinates. All su's are estimated from the variances of the (full) variance-covariance matrix. The cell esds are taken into account in the estimation of distances, angles and torsion angles
Refinement. Refinement of *F*^2^ against ALL reflections. The weighted *R*-factor *wR* and goodness of fit *S* are based on *F*^2^, conventional *R*-factors *R* are based on *F*, with *F* set to zero for negative *F*^2^. The threshold expression of *F*^2^ > σ(*F*^2^) is used only for calculating *R*-factors(gt) *etc*. and is not relevant to the choice of reflections for refinement. *R*-factors based on *F*^2^ are statistically about twice as large as those based on *F*, and *R*- factors based on ALL data will be even larger.

### Fractional atomic coordinates and isotropic or equivalent isotropic displacement parameters (Å^2^)

**Table:**

	*x*	*y*	*z*	*U*_iso_*/*U*_eq_	
O1	1.2543 (2)	0.83264 (13)	0.37167 (12)	0.0880 (8)	
O2	1.2930 (2)	0.83537 (15)	0.25658 (14)	0.0970 (9)	
O3	0.8629 (3)	0.96777 (15)	0.15034 (12)	0.0945 (8)	
O4	0.6274 (2)	0.91507 (15)	0.16287 (13)	0.1024 (9)	
O5	0.80556 (18)	0.67237 (12)	0.42022 (9)	0.0670 (6)	
O6	1.19736 (17)	0.62862 (11)	0.26121 (8)	0.0571 (5)	
O7	1.14127 (16)	0.46411 (10)	0.43867 (8)	0.0542 (5)	
N1	1.2085 (2)	0.82324 (14)	0.30347 (14)	0.0665 (8)	
N2	0.7678 (3)	0.91628 (15)	0.17346 (12)	0.0725 (8)	
N3	0.97818 (18)	0.57160 (12)	0.42827 (9)	0.0438 (5)	
N4	1.16646 (19)	0.54767 (11)	0.35010 (9)	0.0419 (5)	
C1	1.0386 (2)	0.80155 (14)	0.27732 (11)	0.0463 (6)	
C2	0.9841 (3)	0.86114 (15)	0.23618 (12)	0.0535 (7)	
C3	0.8257 (3)	0.85337 (15)	0.21745 (12)	0.0532 (7)	
C4	0.7220 (3)	0.78816 (16)	0.23815 (12)	0.0573 (8)	
C5	0.7796 (2)	0.72705 (16)	0.27701 (12)	0.0523 (7)	
C6	0.9407 (2)	0.73108 (14)	0.29834 (10)	0.0422 (6)	
C7	0.9959 (2)	0.66156 (13)	0.33636 (10)	0.0412 (6)	
C8	0.9205 (2)	0.63875 (14)	0.39476 (11)	0.0442 (6)	
C9	1.0979 (2)	0.52410 (13)	0.40734 (10)	0.0399 (6)	
C10	1.1233 (2)	0.61515 (13)	0.31275 (10)	0.0413 (6)	
O8	0.8547 (2)	0.19291 (16)	0.43667 (11)	0.0935 (8)	
O9	0.8049 (3)	0.06694 (16)	0.33392 (12)	0.1072 (9)	
O10	0.37682 (18)	0.18846 (12)	0.11714 (9)	0.0671 (6)	
O11	0.5594 (2)	0.10258 (12)	0.08857 (9)	0.0751 (6)	
O12	0.64772 (16)	0.31204 (10)	0.07995 (8)	0.0498 (5)	
O13	0.41243 (16)	0.57389 (10)	0.07773 (8)	0.0517 (5)	
O14	0.43352 (18)	0.48055 (12)	0.29694 (8)	0.0619 (6)	
N5	0.8058 (2)	0.15384 (18)	0.36769 (12)	0.0688 (8)	
N6	0.5070 (2)	0.16896 (13)	0.13139 (10)	0.0517 (6)	
N7	0.53330 (17)	0.44455 (11)	0.08224 (9)	0.0391 (5)	
N8	0.42292 (18)	0.52477 (12)	0.18645 (9)	0.0436 (5)	
C11	0.7437 (2)	0.21443 (16)	0.32261 (12)	0.0510 (7)	
C12	0.6703 (2)	0.16833 (15)	0.24722 (12)	0.0487 (7)	
C13	0.6020 (2)	0.22549 (14)	0.20738 (10)	0.0416 (6)	
C14	0.6105 (2)	0.32753 (14)	0.23874 (10)	0.0401 (6)	
C15	0.6879 (2)	0.36932 (16)	0.31568 (11)	0.0514 (7)	
C16	0.7528 (2)	0.31432 (17)	0.35756 (12)	0.0558 (8)	
C17	0.5522 (2)	0.39040 (13)	0.19505 (10)	0.0393 (6)	
C18	0.58097 (19)	0.37731 (13)	0.11825 (10)	0.0376 (6)	
C19	0.4529 (2)	0.51796 (13)	0.11341 (10)	0.0389 (6)	
C20	0.4700 (2)	0.46523 (14)	0.23091 (10)	0.0425 (6)	
N9	0.90805 (19)	0.23135 (12)	0.04844 (9)	0.0474 (5)	
C21	0.9873 (2)	0.28472 (14)	0.00108 (11)	0.0453 (6)	
C22	1.1413 (2)	0.27955 (18)	−0.00719 (12)	0.0605 (8)	
C23	1.2100 (3)	0.3305 (2)	−0.05289 (14)	0.0753 (9)	
C24	1.1257 (3)	0.38406 (19)	−0.08902 (14)	0.0756 (10)	
C25	0.9732 (3)	0.38696 (18)	−0.08093 (14)	0.0700 (9)	
C26	0.9028 (3)	0.33770 (16)	−0.03520 (12)	0.0557 (7)	
C27	0.9959 (3)	0.2525 (2)	0.12914 (14)	0.0719 (9)	
C28	1.0290 (4)	0.3602 (2)	0.17372 (16)	0.0997 (13)	
C29	0.8655 (3)	0.12146 (17)	0.00675 (17)	0.0765 (10)	
C30	0.7452 (4)	0.0983 (2)	−0.06477 (17)	0.1068 (14)	
N10	0.3631 (2)	0.20996 (15)	0.50188 (11)	0.0603 (7)	
C31	0.3160 (3)	0.17238 (16)	0.41624 (13)	0.0587 (8)	
C32	0.3835 (4)	0.10006 (19)	0.37113 (17)	0.0839 (11)	
C33	0.3349 (4)	0.0697 (2)	0.28871 (18)	0.0924 (13)	
C34	0.2279 (5)	0.1152 (3)	0.2591 (2)	0.1064 (16)	
C35	0.1642 (4)	0.1859 (3)	0.30427 (19)	0.1081 (14)	
C36	0.2082 (3)	0.2158 (2)	0.38384 (15)	0.0749 (10)	
C37	0.5166 (3)	0.2857 (2)	0.52637 (16)	0.0776 (10)	
C38	0.5019 (3)	0.3733 (2)	0.49872 (17)	0.0838 (10)	
C39	0.3632 (4)	0.1319 (2)	0.54130 (18)	0.0955 (14)	
C40	0.2051 (5)	0.0682 (2)	0.5247 (2)	0.1146 (18)	
H2	1.05320	0.90540	0.22160	0.0640*	
H3A	0.932 (2)	0.5593 (14)	0.4664 (10)	0.051 (5)*	
H4	0.61460	0.78520	0.22620	0.0690*	
H4A	1.2469 (19)	0.5216 (14)	0.3346 (11)	0.045 (5)*	
H5	0.70910	0.68140	0.28960	0.0630*	
H7A	0.550 (2)	0.4353 (14)	0.0340 (9)	0.045 (5)*	
H8A	0.361 (2)	0.5667 (13)	0.2072 (11)	0.052 (6)*	
H12	0.66660	0.10090	0.22360	0.0580*	
H15	0.69560	0.43710	0.33940	0.0620*	
H16	0.80230	0.34420	0.40890	0.0670*	
H9A	0.8194 (19)	0.2558 (14)	0.0571 (11)	0.051 (6)*	
H22	1.19750	0.24290	0.01730	0.0730*	
H23	1.31390	0.32830	−0.05920	0.0900*	
H24	1.17320	0.41860	−0.11920	0.0910*	
H25	0.91630	0.42240	−0.10640	0.0840*	
H26	0.79900	0.34040	−0.02900	0.0670*	
H27A	0.93530	0.21690	0.15740	0.0860*	
H27B	1.09420	0.22810	0.12550	0.0860*	
H28A	1.08570	0.36990	0.22470	0.1490*	
H28B	0.93200	0.38450	0.17870	0.1490*	
H28C	1.09050	0.39580	0.14650	0.1490*	
H29A	0.95900	0.09610	−0.00800	0.0920*	
H29B	0.82530	0.08810	0.04210	0.0920*	
H30A	0.72210	0.02790	−0.08970	0.1600*	
H30B	0.78490	0.13050	−0.10020	0.1600*	
H30C	0.65140	0.12160	−0.05020	0.1600*	
H10A	0.290 (2)	0.2464 (16)	0.5230 (13)	0.074 (7)*	
H32	0.45870	0.07160	0.39350	0.1000*	
H33	0.37580	0.01960	0.25590	0.1110*	
H34	0.19760	0.09640	0.20530	0.1280*	
H35	0.09000	0.21490	0.28190	0.1300*	
H36	0.16470	0.26560	0.41550	0.0900*	
H37A	0.54380	0.30760	0.58280	0.0930*	
H37B	0.60040	0.25420	0.50480	0.0930*	
H38A	0.59950	0.41900	0.51440	0.1260*	
H38B	0.42080	0.40560	0.52110	0.1260*	
H38C	0.47590	0.35180	0.44280	0.1260*	
H39A	0.39300	0.16300	0.59720	0.1140*	
H39B	0.43990	0.09100	0.52320	0.1140*	
H40A	0.20820	0.01830	0.55050	0.1720*	
H40B	0.17600	0.03700	0.46940	0.1720*	
H40C	0.12950	0.10850	0.54360	0.1720*	

### Atomic displacement parameters (Å^2^)

**Table:**

	*U*^11^	*U*^22^	*U*^33^	*U*^12^	*U*^13^	*U*^23^
O1	0.0757 (12)	0.0802 (12)	0.1037 (15)	0.0067 (9)	−0.0243 (11)	0.0390 (11)
O2	0.0658 (11)	0.1039 (14)	0.160 (2)	0.0252 (10)	0.0406 (12)	0.0874 (15)
O3	0.1146 (16)	0.0906 (13)	0.1059 (15)	0.0314 (12)	0.0118 (12)	0.0690 (12)
O4	0.0853 (14)	0.0965 (14)	0.1368 (18)	0.0291 (11)	−0.0221 (12)	0.0629 (13)
O5	0.0707 (10)	0.0887 (11)	0.0727 (10)	0.0480 (9)	0.0408 (8)	0.0465 (9)
O6	0.0749 (10)	0.0678 (9)	0.0581 (8)	0.0416 (8)	0.0374 (7)	0.0418 (8)
O7	0.0630 (9)	0.0613 (9)	0.0612 (9)	0.0280 (7)	0.0245 (7)	0.0411 (7)
N1	0.0558 (12)	0.0570 (11)	0.0991 (16)	0.0148 (9)	0.0068 (12)	0.0431 (11)
N2	0.0906 (16)	0.0606 (12)	0.0742 (13)	0.0284 (12)	−0.0027 (12)	0.0323 (11)
N3	0.0509 (9)	0.0515 (9)	0.0431 (9)	0.0198 (8)	0.0200 (7)	0.0266 (8)
N4	0.0511 (9)	0.0454 (9)	0.0423 (9)	0.0228 (8)	0.0193 (7)	0.0231 (7)
C1	0.0506 (11)	0.0448 (11)	0.0501 (11)	0.0180 (9)	0.0094 (9)	0.0200 (9)
C2	0.0681 (14)	0.0447 (11)	0.0563 (12)	0.0163 (10)	0.0120 (10)	0.0252 (10)
C3	0.0683 (14)	0.0487 (12)	0.0497 (12)	0.0265 (10)	0.0037 (10)	0.0212 (10)
C4	0.0547 (12)	0.0650 (14)	0.0583 (13)	0.0243 (11)	0.0047 (10)	0.0239 (11)
C5	0.0523 (12)	0.0597 (13)	0.0541 (12)	0.0178 (10)	0.0119 (9)	0.0267 (10)
C6	0.0520 (11)	0.0443 (10)	0.0372 (10)	0.0209 (9)	0.0127 (8)	0.0156 (8)
C7	0.0484 (11)	0.0434 (10)	0.0395 (10)	0.0183 (8)	0.0125 (8)	0.0182 (8)
C8	0.0491 (11)	0.0473 (11)	0.0441 (10)	0.0180 (9)	0.0141 (9)	0.0195 (9)
C9	0.0467 (10)	0.0402 (10)	0.0368 (10)	0.0103 (8)	0.0089 (8)	0.0159 (8)
C10	0.0527 (11)	0.0420 (10)	0.0384 (10)	0.0186 (9)	0.0148 (8)	0.0190 (8)
O8	0.1209 (16)	0.1173 (15)	0.0613 (11)	0.0487 (13)	0.0022 (10)	0.0489 (11)
O9	0.171 (2)	0.0839 (14)	0.0872 (14)	0.0622 (14)	0.0039 (13)	0.0465 (12)
O10	0.0541 (9)	0.0755 (10)	0.0751 (11)	0.0078 (8)	−0.0039 (8)	0.0355 (9)
O11	0.1078 (14)	0.0542 (9)	0.0626 (10)	0.0280 (9)	0.0105 (9)	0.0120 (8)
O12	0.0605 (8)	0.0525 (8)	0.0537 (8)	0.0308 (7)	0.0282 (7)	0.0264 (7)
O13	0.0647 (9)	0.0573 (8)	0.0543 (8)	0.0329 (7)	0.0253 (7)	0.0340 (7)
O14	0.0797 (10)	0.0858 (11)	0.0436 (8)	0.0502 (9)	0.0286 (7)	0.0332 (8)
N5	0.0785 (14)	0.0865 (16)	0.0638 (13)	0.0372 (11)	0.0154 (10)	0.0462 (12)
N6	0.0591 (11)	0.0497 (10)	0.0548 (10)	0.0108 (8)	0.0089 (9)	0.0287 (9)
N7	0.0459 (9)	0.0447 (9)	0.0385 (8)	0.0187 (7)	0.0171 (7)	0.0221 (7)
N8	0.0511 (9)	0.0484 (9)	0.0445 (9)	0.0269 (8)	0.0202 (7)	0.0217 (7)
C11	0.0501 (11)	0.0688 (14)	0.0513 (12)	0.0250 (10)	0.0127 (9)	0.0370 (11)
C12	0.0533 (12)	0.0507 (11)	0.0572 (12)	0.0214 (9)	0.0187 (10)	0.0306 (10)
C13	0.0418 (10)	0.0513 (11)	0.0414 (10)	0.0145 (8)	0.0114 (8)	0.0245 (9)
C14	0.0368 (10)	0.0491 (11)	0.0453 (10)	0.0162 (8)	0.0156 (8)	0.0243 (9)
C15	0.0572 (12)	0.0542 (12)	0.0485 (12)	0.0193 (10)	0.0077 (9)	0.0204 (10)
C16	0.0584 (13)	0.0711 (15)	0.0444 (11)	0.0213 (11)	0.0060 (9)	0.0243 (11)
C17	0.0411 (10)	0.0439 (10)	0.0425 (10)	0.0155 (8)	0.0140 (8)	0.0216 (8)
C18	0.0347 (9)	0.0400 (10)	0.0467 (10)	0.0132 (8)	0.0125 (8)	0.0213 (8)
C19	0.0392 (10)	0.0427 (10)	0.0433 (10)	0.0132 (8)	0.0141 (8)	0.0209 (8)
C20	0.0453 (10)	0.0509 (11)	0.0414 (10)	0.0191 (9)	0.0134 (8)	0.0228 (9)
N9	0.0442 (9)	0.0537 (10)	0.0499 (9)	0.0174 (8)	0.0119 (7)	0.0191 (8)
C21	0.0449 (11)	0.0485 (11)	0.0404 (10)	0.0077 (9)	0.0091 (8)	0.0097 (9)
C22	0.0441 (12)	0.0820 (16)	0.0523 (12)	0.0099 (11)	0.0069 (10)	0.0165 (11)
C23	0.0504 (13)	0.102 (2)	0.0544 (14)	−0.0132 (13)	0.0140 (11)	0.0044 (14)
C24	0.091 (2)	0.0739 (17)	0.0516 (14)	−0.0166 (14)	0.0174 (13)	0.0151 (12)
C25	0.0928 (19)	0.0659 (15)	0.0575 (14)	0.0139 (13)	0.0183 (13)	0.0261 (12)
C26	0.0603 (13)	0.0615 (13)	0.0520 (12)	0.0188 (11)	0.0152 (10)	0.0220 (11)
C27	0.0580 (14)	0.112 (2)	0.0587 (14)	0.0186 (14)	0.0075 (11)	0.0452 (15)
C28	0.091 (2)	0.127 (3)	0.0545 (16)	−0.0118 (18)	−0.0031 (14)	0.0060 (17)
C29	0.0864 (18)	0.0517 (14)	0.101 (2)	0.0179 (13)	0.0414 (16)	0.0259 (14)
C30	0.132 (3)	0.084 (2)	0.0719 (19)	−0.0339 (19)	0.0237 (19)	−0.0054 (16)
N10	0.0578 (11)	0.0746 (13)	0.0574 (11)	0.0316 (10)	0.0166 (9)	0.0225 (10)
C31	0.0650 (14)	0.0558 (13)	0.0570 (13)	0.0094 (11)	0.0233 (11)	0.0153 (11)
C32	0.105 (2)	0.0655 (16)	0.089 (2)	0.0196 (15)	0.0458 (17)	0.0224 (15)
C33	0.122 (3)	0.0617 (17)	0.081 (2)	−0.0132 (17)	0.0552 (19)	0.0007 (15)
C34	0.110 (3)	0.124 (3)	0.072 (2)	−0.017 (2)	0.0238 (19)	0.023 (2)
C35	0.103 (2)	0.158 (3)	0.0690 (19)	0.021 (2)	0.0096 (17)	0.046 (2)
C36	0.0685 (16)	0.0980 (19)	0.0624 (15)	0.0187 (14)	0.0091 (12)	0.0300 (14)
C37	0.0473 (13)	0.105 (2)	0.0712 (16)	0.0150 (13)	0.0035 (11)	0.0143 (15)
C38	0.0696 (16)	0.0881 (19)	0.0866 (19)	−0.0036 (14)	0.0176 (14)	0.0216 (16)
C39	0.122 (3)	0.106 (2)	0.092 (2)	0.059 (2)	0.0330 (18)	0.0587 (18)
C40	0.175 (4)	0.082 (2)	0.110 (3)	0.019 (2)	0.051 (2)	0.0563 (19)

### Geometric parameters (Å, º)

**Table:**

O1—N1	1.217 (3)	C14—C15	1.398 (3)
O2—N1	1.219 (3)	C14—C17	1.460 (3)
O3—N2	1.224 (3)	C15—C16	1.371 (3)
O4—N2	1.216 (3)	C17—C20	1.411 (3)
O5—C8	1.247 (2)	C17—C18	1.405 (2)
O6—C10	1.238 (2)	C12—H12	0.9300
O7—C9	1.232 (2)	C15—H15	0.9300
O8—N5	1.212 (3)	C16—H16	0.9300
O9—N5	1.215 (3)	C21—C22	1.378 (3)
O10—N6	1.222 (2)	C21—C26	1.369 (3)
O11—N6	1.218 (2)	C22—C23	1.385 (4)
O12—C18	1.247 (2)	C23—C24	1.373 (4)
O13—C19	1.226 (2)	C24—C25	1.360 (4)
O14—C20	1.238 (2)	C25—C26	1.376 (3)
N1—C1	1.467 (3)	C27—C28	1.490 (4)
N2—C3	1.463 (3)	C29—C30	1.497 (4)
N3—C8	1.392 (3)	C22—H22	0.9300
N3—C9	1.352 (2)	C23—H23	0.9300
N4—C9	1.354 (2)	C24—H24	0.9300
N4—C10	1.394 (2)	C25—H25	0.9300
N3—H3A	0.885 (18)	C26—H26	0.9300
N4—H4A	0.874 (18)	C27—H27B	0.9700
N5—C11	1.464 (3)	C27—H27A	0.9700
N6—C13	1.469 (2)	C28—H28A	0.9600
N7—C18	1.388 (2)	C28—H28B	0.9600
N7—C19	1.363 (2)	C28—H28C	0.9600
N8—C19	1.356 (2)	C29—H29A	0.9700
N8—C20	1.398 (3)	C29—H29B	0.9700
N7—H7A	0.877 (16)	C30—H30B	0.9600
N8—H8A	0.892 (19)	C30—H30C	0.9600
N9—C27	1.498 (3)	C30—H30A	0.9600
N9—C29	1.508 (3)	C31—C32	1.367 (4)
N9—C21	1.471 (3)	C31—C36	1.362 (4)
N9—H9A	0.902 (18)	C32—C33	1.425 (4)
N10—C39	1.487 (4)	C33—C34	1.353 (5)
N10—C31	1.477 (3)	C34—C35	1.333 (6)
N10—C37	1.538 (3)	C35—C36	1.372 (4)
N10—H10A	0.92 (2)	C37—C38	1.491 (4)
C1—C6	1.398 (3)	C39—C40	1.495 (5)
C1—C2	1.378 (3)	C32—H32	0.9300
C2—C3	1.367 (4)	C33—H33	0.9300
C3—C4	1.371 (3)	C34—H34	0.9300
C4—C5	1.379 (3)	C35—H35	0.9300
C5—C6	1.404 (3)	C36—H36	0.9300
C6—C7	1.463 (3)	C37—H37A	0.9700
C7—C10	1.414 (3)	C37—H37B	0.9700
C7—C8	1.399 (3)	C38—H38A	0.9600
C2—H2	0.9300	C38—H38B	0.9600
C4—H4	0.9300	C38—H38C	0.9600
C5—H5	0.9300	C39—H39A	0.9700
C11—C12	1.371 (3)	C39—H39B	0.9700
C11—C16	1.373 (3)	C40—H40A	0.9600
C12—C13	1.384 (3)	C40—H40B	0.9600
C13—C14	1.393 (3)	C40—H40C	0.9600
			
O1—N1—O2	124.8 (2)	C13—C12—H12	121.00
O1—N1—C1	117.30 (19)	C11—C12—H12	121.00
O2—N1—C1	117.8 (2)	C16—C15—H15	119.00
O3—N2—O4	123.8 (2)	C14—C15—H15	119.00
O3—N2—C3	118.5 (2)	C15—C16—H16	121.00
O4—N2—C3	117.7 (2)	C11—C16—H16	120.00
C8—N3—C9	125.43 (16)	C22—C21—C26	121.5 (2)
C9—N4—C10	125.46 (16)	N9—C21—C26	118.04 (18)
C9—N3—H3A	117.8 (13)	N9—C21—C22	120.46 (18)
C8—N3—H3A	116.8 (13)	C21—C22—C23	118.2 (2)
C9—N4—H4A	120.5 (13)	C22—C23—C24	120.4 (2)
C10—N4—H4A	114.0 (13)	C23—C24—C25	120.4 (2)
O9—N5—C11	118.1 (2)	C24—C25—C26	120.2 (2)
O8—N5—O9	123.6 (2)	C21—C26—C25	119.3 (2)
O8—N5—C11	118.3 (2)	N9—C27—C28	112.7 (2)
O10—N6—C13	117.51 (17)	N9—C29—C30	112.1 (2)
O11—N6—C13	118.07 (17)	C21—C22—H22	121.00
O10—N6—O11	124.30 (18)	C23—C22—H22	121.00
C18—N7—C19	125.40 (15)	C22—C23—H23	120.00
C19—N8—C20	125.59 (16)	C24—C23—H23	120.00
C19—N7—H7A	118.0 (13)	C23—C24—H24	120.00
C18—N7—H7A	116.4 (13)	C25—C24—H24	120.00
C20—N8—H8A	115.6 (12)	C24—C25—H25	120.00
C19—N8—H8A	118.6 (12)	C26—C25—H25	120.00
C21—N9—C29	111.79 (17)	C25—C26—H26	120.00
C27—N9—C29	111.68 (19)	C21—C26—H26	120.00
C21—N9—C27	113.65 (17)	C28—C27—H27B	109.00
C27—N9—H9A	103.2 (12)	N9—C27—H27A	109.00
C21—N9—H9A	107.2 (13)	N9—C27—H27B	109.00
C29—N9—H9A	108.8 (13)	C28—C27—H27A	109.00
C31—N10—C37	112.19 (19)	H27A—C27—H27B	108.00
C31—N10—C39	114.9 (2)	C27—C28—H28A	110.00
C37—N10—C39	112.6 (2)	C27—C28—H28C	110.00
C31—N10—H10A	108.4 (13)	H28B—C28—H28C	109.00
C37—N10—H10A	103.6 (14)	H28A—C28—H28C	109.00
C39—N10—H10A	104.2 (14)	H28A—C28—H28B	109.00
C2—C1—C6	123.47 (18)	C27—C28—H28B	109.00
N1—C1—C6	121.74 (18)	N9—C29—H29B	109.00
N1—C1—C2	114.60 (19)	C30—C29—H29B	109.00
C1—C2—C3	118.2 (2)	H29A—C29—H29B	108.00
C2—C3—C4	121.8 (2)	N9—C29—H29A	109.00
N2—C3—C4	120.0 (2)	C30—C29—H29A	109.00
N2—C3—C2	118.2 (2)	C29—C30—H30C	109.00
C3—C4—C5	118.9 (2)	H30A—C30—H30B	109.00
C4—C5—C6	122.5 (2)	C29—C30—H30B	109.00
C5—C6—C7	120.33 (18)	H30A—C30—H30C	109.00
C1—C6—C7	124.46 (16)	H30B—C30—H30C	110.00
C1—C6—C5	115.15 (18)	C29—C30—H30A	109.00
C6—C7—C8	120.21 (16)	N10—C31—C32	121.1 (2)
C8—C7—C10	119.71 (17)	N10—C31—C36	117.4 (2)
C6—C7—C10	120.06 (16)	C32—C31—C36	121.5 (2)
O5—C8—N3	116.00 (17)	C31—C32—C33	117.8 (3)
N3—C8—C7	117.37 (16)	C32—C33—C34	118.7 (3)
O5—C8—C7	126.63 (19)	C33—C34—C35	122.4 (3)
O7—C9—N3	122.01 (17)	C34—C35—C36	119.9 (3)
N3—C9—N4	115.19 (17)	C31—C36—C35	119.7 (3)
O7—C9—N4	122.80 (17)	N10—C37—C38	111.4 (2)
O6—C10—C7	125.34 (18)	N10—C39—C40	111.0 (3)
N4—C10—C7	116.81 (16)	C31—C32—H32	121.00
O6—C10—N4	117.84 (17)	C33—C32—H32	121.00
C1—C2—H2	121.00	C32—C33—H33	121.00
C3—C2—H2	121.00	C34—C33—H33	121.00
C5—C4—H4	121.00	C33—C34—H34	119.00
C3—C4—H4	121.00	C35—C34—H34	119.00
C4—C5—H5	119.00	C34—C35—H35	120.00
C6—C5—H5	119.00	C36—C35—H35	120.00
C12—C11—C16	121.7 (2)	C31—C36—H36	120.00
N5—C11—C12	118.6 (2)	C35—C36—H36	120.00
N5—C11—C16	119.67 (19)	N10—C37—H37A	109.00
C11—C12—C13	117.8 (2)	N10—C37—H37B	109.00
N6—C13—C14	121.80 (17)	C38—C37—H37A	109.00
C12—C13—C14	123.33 (17)	C38—C37—H37B	109.00
N6—C13—C12	114.66 (18)	H37A—C37—H37B	108.00
C13—C14—C17	124.02 (16)	C37—C38—H38A	110.00
C13—C14—C15	115.49 (18)	C37—C38—H38B	110.00
C15—C14—C17	120.40 (18)	C37—C38—H38C	109.00
C14—C15—C16	122.6 (2)	H38A—C38—H38B	109.00
C11—C16—C15	119.02 (19)	H38A—C38—H38C	109.00
C18—C17—C20	120.48 (17)	H38B—C38—H38C	109.00
C14—C17—C18	119.84 (16)	N10—C39—H39A	109.00
C14—C17—C20	119.66 (16)	N10—C39—H39B	109.00
O12—C18—N7	117.00 (16)	C40—C39—H39A	110.00
N7—C18—C17	116.90 (16)	C40—C39—H39B	109.00
O12—C18—C17	126.09 (17)	H39A—C39—H39B	108.00
N7—C19—N8	115.13 (16)	C39—C40—H40A	109.00
O13—C19—N8	122.97 (17)	C39—C40—H40B	109.00
O13—C19—N7	121.90 (16)	C39—C40—H40C	109.00
N8—C20—C17	116.36 (15)	H40A—C40—H40B	110.00
O14—C20—C17	125.60 (19)	H40A—C40—H40C	109.00
O14—C20—N8	118.01 (17)	H40B—C40—H40C	109.00
			
O1—N1—C1—C2	130.8 (2)	C2—C3—C4—C5	−1.8 (3)
O2—N1—C1—C2	−45.6 (3)	C3—C4—C5—C6	2.0 (3)
O1—N1—C1—C6	−44.4 (3)	C4—C5—C6—C7	−177.22 (19)
O2—N1—C1—C6	139.1 (2)	C4—C5—C6—C1	0.0 (3)
O3—N2—C3—C4	−174.1 (2)	C1—C6—C7—C8	141.06 (19)
O3—N2—C3—C2	5.3 (3)	C5—C6—C7—C8	−42.0 (3)
O4—N2—C3—C2	−173.9 (2)	C1—C6—C7—C10	−40.6 (3)
O4—N2—C3—C4	6.8 (3)	C5—C6—C7—C10	136.35 (19)
C8—N3—C9—O7	−178.71 (18)	C10—C7—C8—N3	1.3 (3)
C8—N3—C9—N4	1.6 (3)	C10—C7—C8—O5	−178.59 (19)
C9—N3—C8—C7	−2.3 (3)	C8—C7—C10—O6	179.07 (18)
C9—N3—C8—O5	177.59 (18)	C8—C7—C10—N4	0.1 (3)
C9—N4—C10—C7	−0.8 (3)	C6—C7—C8—N3	179.61 (17)
C10—N4—C9—O7	−179.67 (17)	C6—C7—C8—O5	−0.2 (3)
C10—N4—C9—N3	0.0 (3)	C6—C7—C10—N4	−178.22 (16)
C9—N4—C10—O6	−179.84 (18)	C6—C7—C10—O6	0.7 (3)
O8—N5—C11—C12	−171.88 (19)	N5—C11—C12—C13	175.26 (17)
O9—N5—C11—C12	7.8 (3)	N5—C11—C16—C15	−177.10 (17)
O9—N5—C11—C16	−175.5 (2)	C16—C11—C12—C13	−1.3 (3)
O8—N5—C11—C16	4.8 (3)	C12—C11—C16—C15	−0.6 (3)
O10—N6—C13—C12	132.40 (19)	C11—C12—C13—C14	3.0 (3)
O10—N6—C13—C14	−42.5 (3)	C11—C12—C13—N6	−171.77 (17)
O11—N6—C13—C14	141.2 (2)	C12—C13—C14—C17	173.92 (18)
O11—N6—C13—C12	−43.9 (3)	C12—C13—C14—C15	−2.7 (3)
C19—N7—C18—C17	−4.2 (3)	N6—C13—C14—C15	171.77 (17)
C19—N7—C18—O12	176.98 (17)	N6—C13—C14—C17	−11.6 (3)
C18—N7—C19—N8	1.7 (3)	C13—C14—C15—C16	0.7 (3)
C18—N7—C19—O13	−178.88 (17)	C15—C14—C17—C18	133.29 (19)
C20—N8—C19—O13	−177.98 (18)	C13—C14—C17—C20	138.12 (19)
C19—N8—C20—C17	−1.7 (3)	C17—C14—C15—C16	−176.07 (17)
C19—N8—C20—O14	180.00 (17)	C13—C14—C17—C18	−43.1 (3)
C20—N8—C19—N7	1.4 (3)	C15—C14—C17—C20	−45.5 (3)
C21—N9—C27—C28	56.7 (3)	C14—C15—C16—C11	0.9 (3)
C29—N9—C27—C28	−175.7 (2)	C14—C17—C20—O14	−4.1 (3)
C27—N9—C21—C22	52.3 (3)	C18—C17—C20—O14	177.14 (19)
C27—N9—C29—C30	165.7 (2)	C20—C17—C18—O12	−177.63 (18)
C29—N9—C21—C22	−75.2 (2)	C18—C17—C20—N8	−1.0 (3)
C27—N9—C21—C26	−128.8 (2)	C14—C17—C20—N8	177.70 (16)
C29—N9—C21—C26	103.7 (2)	C14—C17—C18—O12	3.7 (3)
C21—N9—C29—C30	−65.7 (3)	C20—C17—C18—N7	3.7 (3)
C37—N10—C31—C36	−97.4 (3)	C14—C17—C18—N7	−175.01 (16)
C39—N10—C37—C38	−168.8 (2)	C26—C21—C22—C23	0.6 (3)
C31—N10—C39—C40	−57.8 (3)	N9—C21—C26—C25	−178.91 (19)
C31—N10—C37—C38	59.8 (3)	C22—C21—C26—C25	0.0 (3)
C37—N10—C31—C32	79.7 (3)	N9—C21—C22—C23	179.5 (2)
C39—N10—C31—C36	132.4 (3)	C21—C22—C23—C24	−0.3 (4)
C39—N10—C31—C32	−50.6 (3)	C22—C23—C24—C25	−0.6 (4)
C37—N10—C39—C40	172.2 (2)	C23—C24—C25—C26	1.2 (4)
N1—C1—C6—C5	172.57 (19)	C24—C25—C26—C21	−0.9 (4)
C2—C1—C6—C5	−2.3 (3)	N10—C31—C32—C33	−178.3 (3)
N1—C1—C6—C7	−10.4 (3)	C36—C31—C32—C33	−1.3 (4)
C6—C1—C2—C3	2.5 (3)	N10—C31—C36—C35	177.9 (3)
C2—C1—C6—C7	174.82 (19)	C32—C31—C36—C35	0.8 (4)
N1—C1—C2—C3	−172.69 (19)	C31—C32—C33—C34	1.6 (5)
C1—C2—C3—N2	−179.72 (19)	C32—C33—C34—C35	−1.3 (6)
C1—C2—C3—C4	−0.3 (3)	C33—C34—C35—C36	0.8 (6)
N2—C3—C4—C5	177.6 (2)	C34—C35—C36—C31	−0.5 (5)

### Hydrogen-bond geometry (Å, º)

**Table:**

*D*—H···*A*	*D*—H	H···*A*	*D*···*A*	*D*—H···*A*
N3—H3*A*···O7^i^	0.89 (2)	2.00 (2)	2.878 (2)	172 (2)
N4—H4*A*···O14^ii^	0.87 (2)	1.93 (2)	2.802 (2)	172 (2)
N7—H7*A*···O13^iii^	0.88 (2)	2.06 (2)	2.931 (2)	175 (2)
N8—H8*A*···O6^iv^	0.89 (2)	1.98 (2)	2.852 (2)	164 (2)
N9—H9*A*···O12	0.90 (2)	1.83 (2)	2.726 (2)	176 (1)
N10—H10*A*···O5^v^	0.92 (2)	1.69 (2)	2.598 (3)	166 (2)
C12—H12···O4^vi^	0.93	2.52	3.451 (3)	174
C26—H26···O12	0.93	2.59	3.272 (3)	131
C26—H26···O13^iii^	0.93	2.56	3.281 (3)	135
C29—H29*B*···O11	0.97	2.57	3.215 (3)	124
C38—H38*A*···O7^i^	0.96	2.52	3.484 (3)	177

Symmetry codes: (i) −*x*+2, −*y*+1, −*z*+1; (ii) *x*+1, *y*, *z*; (iii) −*x*+1, −*y*+1, −*z*; (iv) *x*−1, *y*, *z*; (v) −*x*+1, −*y*+1, −*z*+1; (vi) *x*, *y*−1, *z*.
